# The ATP-Binding Cassette Proteins of the Deep-Branching Protozoan Parasite *Trichomonas vaginalis*

**DOI:** 10.1371/journal.pntd.0001693

**Published:** 2012-06-19

**Authors:** Christopher Kay, Katharine D. Woodward, Karen Lawler, Tim J. Self, Sabrina D. Dyall, Ian D. Kerr

**Affiliations:** 1 School of Biomedical Sciences, University of Nottingham Medical School, Queen's Medical Centre, Nottingham, United Kingdom; 2 Department of Biosciences, University of Mauritius, Reduit, Mauritius; University of Pittsburgh, United States of America

## Abstract

The ATP binding cassette (ABC) proteins are a family of membrane transporters and regulatory proteins responsible for diverse and critical cellular process in all organisms. To date, there has been no attempt to investigate this class of proteins in the infectious parasite *Trichomonas vaginalis*. We have utilized a combination of bioinformatics, gene sequence analysis, gene expression and confocal microscopy to investigate the ABC proteins of *T. vaginalis*. We demonstrate that, uniquely among eukaryotes, *T. vaginalis* possesses no intact full-length ABC transporters and has undergone a dramatic expansion of some ABC protein sub-families. Furthermore, we provide preliminary evidence that *T. vaginalis* is able to read through in-frame stop codons to express ABC transporter components from gene pairs in a head-to-tail orientation. Finally, with confocal microscopy we demonstrate the expression and endoplasmic reticulum localization of a number of *T. vaginalis* ABC transporters.

## Author Summary

The parasite *Trichomonas vaginalis* infects in excess of 100 million people per year, and is a contributory factor to enhanced transmission rates of HIV, the causative virus in AIDS. As such, *T. vaginalis* infection is an important public health concern. Understanding the biology of the organism is important to determine aspects of the response to drug treatment, host:parasite interactions and so on. We have investigated an important family of proteins – the ATP binding cassette transporters – which are present in the membranes of all cells, and which contribute to a diverse spectrum of important cellular processes. The ABC transporters of *T. vaginalis* were identified by analysis of primary amino acid sequence data, and examined by subsequent protein and gene expression studies. Our most important conclusion is that – uniquely amongst eukaryotes - *T. vaginalis* has no ABC transporters capable of acting as monomers. In other words, its ABC transporters must all act by forming functional complexes with other ABC proteins. This has implications for our understanding not just of the parasite's biology, but also its evolution. In summary our analysis opens up the path for future research of individual members of the ABC protein family in *T. vaginalis*.

## Introduction

*Trichomonas vaginalis* is a human-infective parasitic protozoan that is the most prevalent causative agent for non-viral sexually transmitted infections, with an estimated annual incidence of at least 170 million new cases of trichomoniasis worldwide [1]. Characteristic of other parabasalids in the order Trichomonadida, *T. vaginalis* is a flagellated, microaerophilic eukaryote which displays many features typical of eukaryotic cells including a membrane-bound nucleus, endoplasmic reticulum, ribosomes and the nine plus two arrangement of microtubules [2]. The unicellular organism possesses five microtubule-based flagella – four located anteriorly and one posteriorly associated with an undulating membrane, the structure responsible for the parasite's distinctive quivering movement [3]. An additional key morphological feature of *T. vaginalis* is its axostyle, a cylindrical structure spanning the length of the cell from an anteriorly positioned nucleus. Also composed of microtubules, it is believed to play a role in anchoring the parasite to the vaginal epithelium [3]. In common with many unicellular eukaryotes with apparently early evolutionary divergence, *T. vaginalis* has a reduced complement of organelles in comparison with higher eukaryotes, lacking both mitochondria and peroxisomes, but containing instead a hydrogen producing organelle (the hydrogenosome) which may be a relic of an endosymbiotically acquired mitochondrion-like organelle, or which may be the result of a 2^nd^ symbiotic event (see [4] for a review).

Given the unusual cell biology of *T. vaginalis* we decided to investigate a class of membrane proteins (ATP binding cassette (ABC) transporters) localized to the plasma membrane and organellar membranes of all eukaryotic cells, and examine differences in the complement of these proteins in *T. vaginalis* compared to other eukaryotes, with a longer term view to determining the contribution of ABC transporters to the parasite's biology.

ATP binding cassette (ABC) systems encompass a family of proteins found in all 3 domains of life, which are responsible for an abundance of transport roles as well as a variety of intracellular non-transport processes including gene regulation and DNA repair [5]. The vast majority of ABC transporters in eukaryotes are exporters, whilst prokaryotes encode functioning importers too. All proteins in this family are defined by the presence of a highly conserved nucleotide binding domain (NBD; the eponymous ATP binding cassette (ABC)), an ATPase domain with characteristic motifs including a Walker A, Walker B and Signature sequences, which contribute to the hydrolysis of ATP, the energy of which drives the various cellular processes mentioned. The NBDs are highly conserved among different ABC proteins, sharing typically greater than 25% sequence identity [6], and a common 3-dimensional fold [6].

In addition to the cytoplasmic NBDs, ABC transporters contain transmembrane domains (TMDs), which span the membrane numerous times via α helices (typically 4–11 helices per domain, [7]), and which contain binding sites for transported substrates. The typical configuration for a functional ABC transporter is believed to comprise 2 NBDs and 2 TMDs, although these need not necessarily be present within the same polypeptide. For example, “full-transporters” are defined as containing all four domains within the same polypeptide, whilst “half-transporters” typically comprise a single NBD and TMD within one polypeptide and are believed to either homo- or hetero-dimerize in order to function [5] [8]. In contrast to the NBDs, the TMD components show considerable sequence variation between unrelated transporters reflecting their role in recognition and transport of substrate.

Based on the organisation of their NBDs and TMDs as well as features such as membrane topology, sequence homology and gene structure, eukaryotic ABC proteins have been grouped into 7 subfamilies - ABCA to ABCG [7], [8], although occasionally sequences have been identified as not fitting with this classification and these have been annotated as ABCH and ABCI sequences (e.g. see [9]). The availability of the recently completed *T. vaginalis* genome sequence [10] enabled a database search for ABC proteins within *T. vaginalis* to be conducted, as presented here, using the sequences of known ABC proteins from other species for comparison. A phylogenetic classification of the hypothetical ABC proteins into subfamilies has been conducted, accompanied by discussion regarding the putative location and function of different transporters within the parasite. In addition we have performed sub-cellular localization studies on selected ABC proteins to validate our analysis. Finally, comparisons are drawn between the ABC families of *T. vaginalis* and three other sequenced protists *P. falciparum, E. histolytica* and *G. lamblia* [11], [12], [13].

## Materials and Methods

### Cell cultivation and transfection

The sequenced *T. vaginalis* strain G3 (ATCC PRA-98; [10]), kindly provided by G. H. Coombs, was used for genomic DNA preparation throughout this study and was grown in modified TYM medium [14]. Strain C1 (ATCC 30001), which is less pathogenic, was employed for transfection and localization studies.

### Transfection of *T. vaginalis*

Late stage cultures of *T. vaginalis* C1 were centrifuged (1500 g, 10 minutes, 4°C) and resuspended in supplemented Diamond's medium to a density of 10^8^ cells/ml. Cells (3×10^7^) were incubated with plasmid DNA (50 µg) for 15 minutes on ice and then electroporated 350 V, 960 µF (BioRad GenePulser) in chilled 0.4 cm spacing electroporation cuvettes (GeneFlow). Electroporated cells were immediately diluted into 50 ml of complete media, pre-warmed to 37°C and then incubated for a further 4 hours, prior to the addition of G418 to 50 µg/ml, and left overnight, before surviving cells (those in motile suspension) were transferred into fresh complete, selective media and incubated for 3–21 days until a density of ca. 2×10^6^ cells/ml was obtained, whereupon they were passaged as described above.

### Extraction of nucleic acids from *T. vaginalis*

Genomic DNA (gDNA) was isolated from 3×10^8^ *T. vaginalis* cells as described previously [15] and resuspended in 500 µl TE containing 10 µg/ml RNase A. Total RNA was extracted from 2×10^9^ *T. vaginalis* cells by the single-step acid guanidinium thiocyanate-phenol-chloroform method [16]. 1 mg of total RNA was processed using the PolyATtract mRNA isolation system (Promega Corporation, Madison, WI, USA) according to manufacturers' instructions to yield ca. 10 µg of enriched polyA+ RNA that was stored at −80°C until required.

### Reverse transcription and PCR

Reverse transcription and PCR of specific regions of the genes TVAG_275410, TVAG_275420, TVAG_072410, TVAG_072420 and TVAG_470720 were carried out according to manufacturer's instructions using the Access RT-PCR System (Promega Corporation, Madison, WI, USA) with appropriate primers listed in Table S1. Bands of interest were separated by agarose gel electrophoresis, and DNA was extracted using the Qiaquick Gel Extraction Kit (Qiagen Inc.) prior to treatment with T4 DNA polymerase (New England Biolabs) to remove 3′ A overhangs introduced by *Tfl* DNA polymerase in the Access RT-PCR System. To allow for sequencing, the blunt-ended fragments were then ligated into the pSC-B vector using the StrataClone Blunt PCR Cloning Kit (Stratagene). Where indicated, specific gene fragments were amplified by the polymerase chain reaction (PCR) from *T. vaginalis* gDNA using Phusion DNA polymerase (New England Biolabs, Inc.) according to manufacturer's recommendations.

### Construction of plasmids for transfection of *T. vaginalis*

cDNA regions comprising the open reading frames for genes *TVAG_470720*, *TVAG_605460* and fused *TVAG_415980/TVAG_415990* were amplified by PCR using the primer pairs 470720NdeI and 470720BamHI, 605460NdeI and 605460BamHI, 415980NdeI and 415990BamHI respectively (Table S1). The introduced NdeI and BamHI restriction recognition sites facilitated ligation into corresponding sites on the pTagVag2 vector, thereby resulting in C-terminal tagging with a double haemagluttinin epitope [17], (a kind gift of Professor Jan Tachezy). The resulting plasmid constructs, pTagVag2-470720, pTagVag2-605460 and pTagVag2-415980/90 were maintained in *E. coli* XL1-Blue and plasmid DNA was purified using Qiagen maxiprep kits. The purified plasmid DNA was further subjected to ethanol precipitation and resuspended in water under sterile conditions at a concentration of 10 µg/µl for transfection into *T. vaginalis*.

### Confocal microscopy

Late stage cells were centrifuged (1500 g, 10 minutes, 4°C) and resuspended in phosphate buffered saline (PBS) at 1×10^7^/ml. Aliquots (0.5 ml) of this suspension were layered onto silane covered microscope slides (Sigma) and left to adhere for 30 minutes at room temperature. Non-adherent cells were removed by washing once with PBS, and remaining cells were fixed and permeabilized with 0.5 ml 4% w/v paraformaldehyde, 0.1% v/v Triton X-100 for 20 minutes at room temperature. Slides were washed twice with PBS, and then incubated for 1 hour at room temperature in blocking solution (PBS supplemented with 0.25% w/v each BSA and fish gelatin). Slides were then incubated with primary antibody (mouse anti-HA, 1∶2500 or rabbit anti-BiP, 1∶1000 in blocking solution) for 1 hour at room temperature, washed twice with PBS and then incubated with secondary antibody (species specific AlexaFluor-488, 1∶1000 in blocking solution) for 1 hour at room temperature in the dark. After further washing in PBS, slides were treated with RNAase (100 µg/mL in PBS, 37°C, 20 minutes), washed, and nuclei stained by addition of propidium iodide (3.3 µg/mL in PBS, 5 minutes) and then mounted with 50% v/v glycerol in PBS. Slides were kept at 4°C in the dark until analyzed, and could be stored for at least 2 months without loss of signal quality. Slides were analysed on a Zeiss LSM 710 confocal laser scanning microscope, using a 63× oil-immersion objective. The fluorescent tags were excited using a laser sources at 488 nm (Alexa488) and 561 nm (propidium iodide), and emitted light collected. Image files were subsequently processed using the Zeiss LSM Image Browser software.

### Bioinformatics analyses

The protein sequence of human ABCB1/P-glycoprotein (AAA59575) was used as a query for a homology search of TrichDB (http://trichdb.org/trichdb), the complete *T. vaginalis* genome database, using BLASTp. Pairs of sequences considered redundant due to greater than 95% amino acid identity were removed prior to further analysis. All remaining sequences were screened manually for Walker A (GxxGxGK(S/T), where x = any amino acid), Walker B (hhhhDE, where h = hydrophobic amino acid) and ABC signature (LSGGQ) motifs. Putative TM spanning regions of hypothetical *T. vaginalis* ABC transporters was predicted using the programs TMHMM [18], TopPred [19], and HMMTOP [20].

Multiple BLASTp searches were performed on the NCBI (National Centre for Biotechnology Information) and UNIPROT websites to identify characterised and curated homologues of the hypothetical ABC proteins of *T. vaginalis* in other species and thus facilitate classification of the *T. vaginalis* proteins. Each protein sequence was also used as a query to search the Expressed Sequence Tag (EST) database using tBLASTn. The EST database consists of 26,491 single pass cDNA sequences obtained from the C1 and T1 strains of *T. vaginalis* (TrichDB). Protein sequences showing greater than 97% identity to translated ESTs were categorised as being expressed in *T. vaginalis*.

Consensus phylogenetic trees were constructed via a multistep process to examine relationships between different protein sequences. Multiple sequence alignment of the hypothetical ABC proteins was performed on Bioedit with ClustalW_2 using the BLOSUM-62 matrix. Alignments were manually edited to remove internal gaps and N and C-terminal extensions where necessary to prevent differences in sequence length affecting protein clustering. The amended alignment was bootstrapped with 500 replications using Seqboot, whilst Protpars generated trees from the resulting alignments to be used by Consense in producing a consensus. Seqboot, Protpars and Consense all form part of the Phylip Package Version 4.0, which was accessed via the Mobyle website (http://mobyle.pasteur.fr). Trees were visualised using Treeview.

## Results and Discussion

### *T. vaginalis* has 98 putative ABC proteins

A BLASTp search of TrichDB [21] using human P-glycoprotein [22] as a query sequence identified 102 predicted *T. vaginalis* ABC proteins, four of which showed >95% identity to another sequence and so were removed to avoid redundancy (TVAG_059100, TVAG_132360, TVAG_431960 and TVAG_510260). The 98 hypothetical ABC proteins identified here exceeds the 88 originally estimated based on the draft genome sequence [10] and, compared with the number of ABC proteins identified in other species, constitutes a significant total. Table 1 compares the ABC family of *T. vaginalis* with multi- and unicellular non-parasitic species as well as four other disease-causing parasites: *P. falciparum, E. histolytica, L. major* and *G. lamblia*. The number of ABC genes in *T. vaginalis* exceeds all but the two plant species [9], [23], and indicates that the ABC family of genes has undergone considerable expansion in *T. vaginalis*. Genome analysis indicates many other gene families (including several involved in membrane trafficking and transport) have expanded similarly [10]. As with these, expression proteomics under diverse growth conditions is required before the tags “putative” or “hypothetical” can be dispensed with. The large number of ABC proteins in plants is believed to be partly due to genome expansion and also due to functional diversification [9]. The putative functional diversity of *T. vaginalis* ABC proteins will be discussed below.

**Table 1:** ABC proteins in sequence genomes.

Organism	Estimate number of proteins	Estimated number of ABC proteins	Reference
*T. vaginalis*	60000	98^2^	This work
*H. sapiens*	25000	48^2^	[7]
*C. elegans*	18400	60	[42]
*A. thaliana*	35000	129^2^	[9]
*O. sativa*	37500	121	[9]
*S. cerevisiae*	6300	29^2^	[43]
*L. major*	8500	42	[44]
*P. falciparum*	5300	16^1^	This work & [31]
*E. histolytica*	9900	26^1^	This work & [31]
*G. lamblia*	6500	22^2^	This work & [31]
*E. coli*	4300	79^2^	[45]
*B. subtilis*	4100	84^2^	[46]

1 these numbers are greater than those presented in a recent analysis of ABC transporters from protozoan parasites [31].

2 The proportion of full length ABC transporter genes is 0% in *T. vaginalis*, 69% in humans, 62% in *Arabidopsis*, 83% in yeast, 68% in *Giardia* and 0% in *E. coli* and *B. subtilis*.

The lengths of hypothetical proteins varied from 116 to 919 amino acids, although for three sequences this is difficult to verify as three are located at the ends of unassembled sequence scaffolds (TVAG_241640, TVAG_542450 and TVAG_542470). All 98 sequences were analysed manually for the presence of Walker A (GxxGxGKS/T), Walker B (hhhhDE) and Signature motifs (LSGGQ). The majority were found to contain all three although it was common in putative ABC proteins for one of these, usually the Signature, to be very distinct from the canonical sequences (Table S2). This is not atypical as examination of multiple ABC transporter families has previously shown (e.g. [24]). Analysis of the hydrophobicity of the ABC proteins using TOPPRED, TMHMM and HMMTOP resulted in predictions of between 0 to 9 transmembrane (TM) helices. Indeed 26 sequences were identified as containing no transmembrane regions, a number far in excess of any other non-plant, eukaryotic genome. No single sequence appeared to encode two blocks of multiple TMHs, separated by an NBD sequence, indicating that the *T. vaginalis* genome does not encode any full length transporters, an observation discussed further below. Based on the number and order of NBDs and TMDs, the topology of each protein was defined and table S2 presents an inventory of all 98 predicted proteins with respect to length, membrane topology, motifs and subfamily classification (explained below).

### Classification of ABC proteins of *T. vaginalis*

**Figure 1:**
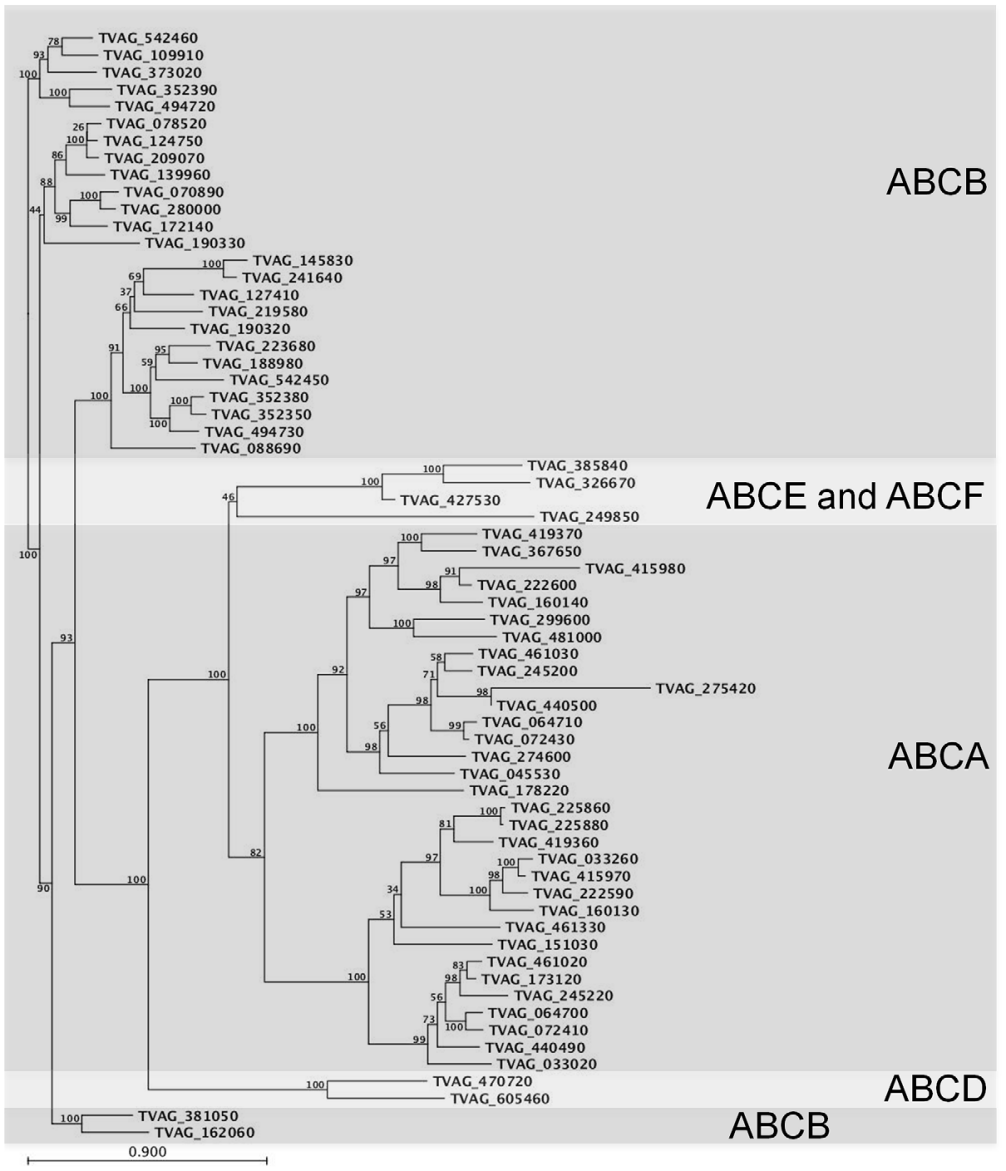
NBDs of 65 predicted ABC proteins of *T. vaginalis*. Multiple sequence alignment and boot-strapping was performed as described in the material and methods. The boot-strap values (percentages) are displayed on all branches. The clustering of the ABC genes from families A–G is evident. The ABCE and F families of non-transporter proteins are highlighted on a lighter background for clarity.

Initial phylogenetic analyses (data not shown) of the *T. vaginalis* ABC protein sequences resulted in a tentative assignment of the majority to one of the major sub-families of ABC proteins documented in eukaryotes. However, bootstrap analyses indicated low certainty in many of the branch-points and thus three further measures were taken to reinforce our assignment of proteins to the ABC sub-families as listed in Table S2. Firstly, we considered the topology of each predicted protein. For example, sequences with a large (>250 amino acids long) predicted extracellular loop (ECL) sandwiched between the first two predicted TM helices were candidates for the ABCA family (e.g. TVAG_173120) as this insertion is exclusively found in ABCA sequences [25]. Secondly, sequences with two consecutive NBDs and no TMDs were likely to represent members of the ABCE or ABCF sub-families (e.g. TVAG_385840) as again in eukaryotes this domain organization is only found in non-transporting ABC proteins [26]. Thirdly, for each sequence we performed BLASTp analyses against the GenBank non-redundant protein sequence database and used the highest ranked sequences as a guide for sub-family allocation. For example, in the case of the TVAG_542450 sequence, which had been previously categorised as being the parasite's homologue of P-glycoprotein/ABCB1 [27], we found that all of the highest-ranking 100 sequences for TVAG_542450 were classified as being predicted or characterised members of the ABCB family. Finally, to improve the accuracy of bootstrap analyses, we removed the confounding factor of highly variable sequence lengths and aligned the NBD sequences only. This analysis demonstrated clear clustering of the ABC proteins into several sub-families, and the removal of putative ABCH and ABCI sequences (Table S2) from the analysis further improved the clarity of the sub-classification (Figure 1). The final predicted numbers of sequences in each sub-family are given in Table 1, with numbers from other eukaryotes given for comparison. Among the findings we discuss below are the absence of two families – namely ABCG and ABCC, the expansion of the ABCA sub-family, and the preponderance (31 in total) of proteins that are unclassifiable with the ABCA-ABCG proteins.

### A large number of orphan abc genes in *T. vaginalis*

**Figure 2:**
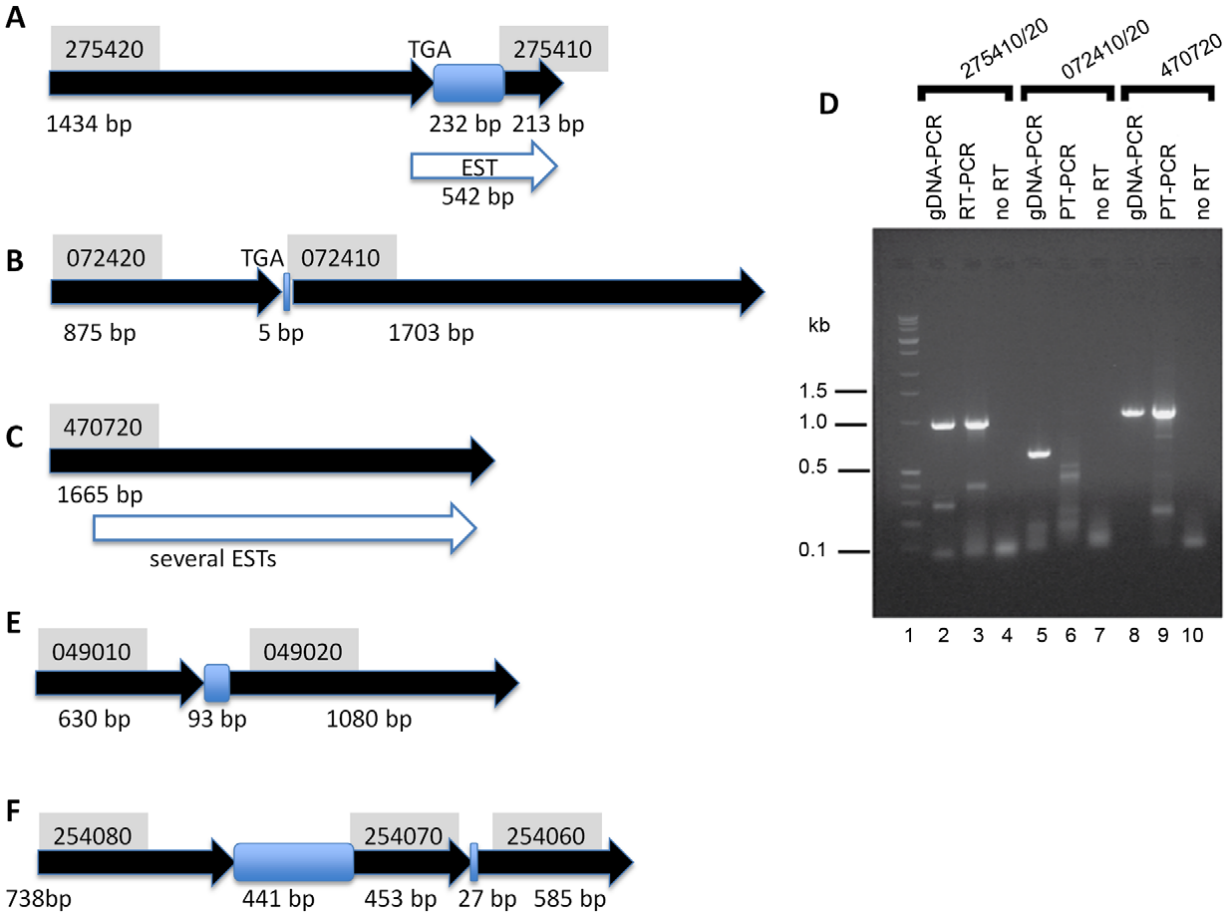
Validation of ABC transporter gene sequence and transcripts from *T. vaginalis*. A, B, C, E, and F. The genomic organisation of 5 combinations of open reading frames (except C, where a single gene is shown) are displayed such that predicted genes are in thick, filled arrows, with the gene identifier on a grey boxed background above, and the gene size in plain font below. The presence of intervening sequences with high homology to other ABC genes elsewhere in the genome is denoted by blue boxes. Expressed sequence tags (ESTs) are indicated by open arrows below the gene of interest. (D) RT-PCR of representative *T. vaginalis* ABC genes. Lanes 2–4: 275410/420; lanes 5–7: 072410/420; lanes 8–10: 470720. Primers for amplification from cDNA are listed in Table S1. Controls lanes (2, 5, 8) employed genomic DNA as template for the PCR, lanes 3, 6 and 9 were from complete reverse transcription and PCR reactions, lanes 4, 7 and 10 lacked the RT enzyme. Novagen Perfect DNA markers are in lane 1.

**Figure 3:**
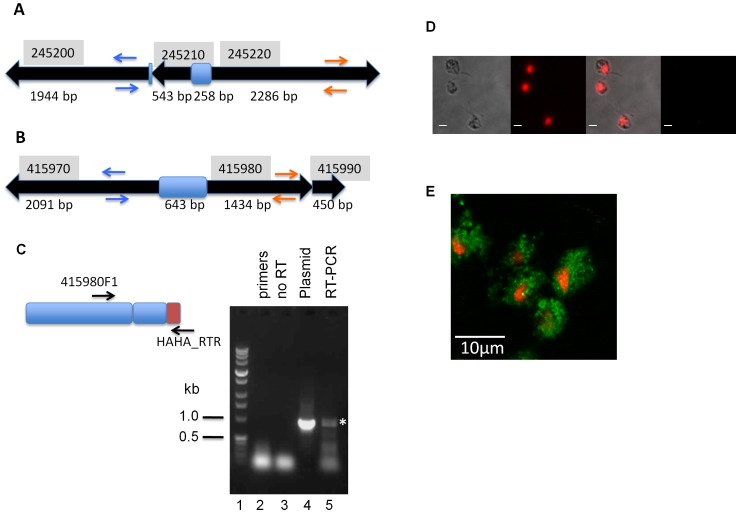
The absence of full length transporters from *T. vaginalis*. **A, B** genomic context of combinations of open reading frames that could encode for an intact full-length ABC transporter. Formatting is as described in Figure 2, with pairs of primers used to verify the genomic organization displayed as blue and orange arrows (Table S1). **C** The two genes TVAG_415980 and TVAG_415990 are separated only by an in-frame stop codon (TGA). RT-PCR analysis of mRNA with primers (black arrows) demonstrates that transcript containing both TVAG_415980 and TVAG_415990 sequences exists (lane 5, asterisk, at the same size as the genomic DNA control). Reactions lacking the RT step (lane 3), or containing primers only (lane 2) verify the specificity of the band in lane 5. **D** Confocal microscopy of *T. vaginalis* C1 cells. The four panels represent (left to right, scale bars 10 µm) bright-field images, detection of nuclear material by propidium iodide staining, overlay of the first pair, and finally, the lack of any anti-HA reactive signal in C1 cells. **E** Confocal microscopy of *T. vaginalis* C1 transformed with a plasmid containing the genomic DNA of TVAG_415980 and TVAG_415990 with the stop codon of the latter replaced by a double haemagluttinin (HA) tag. Parasites were fixed as described in the Methods, examined with a Zeiss LSM 710 confocal microscope, and visualization of HA-tagged ABC TVAG_415980_90 followed incubation with anti-HA primary and an Alexaflour-488 secondary antibody (green).

Upon examination of the chromosomal localisation of the genes listed in Table S2, we noted in excess of 20 examples of ORFs linked on the same loci. Several of these ORFs apparently encode half-transporters with a complete NBD and several transmembrane segments, but a large proportion (see Table S2, “Others”) encoded only part of the NBD on one ORF, and the rest on adjacent genes with a linked head-to-tail orientation (e.g. see Figure 2 and 3). The intergenic regions in the latter cases were found to be relatively small, ranging from 0 to a few hundred bases. BLASTx searches with these intervening sequences revealed that they are themselves highly similar to coding *abc* gene sequences, but were either out of frame with the flanking partial *abc* ORFs, or were in frame but interrupted by stop codons.

To audit whether these *abc* ORFs are genuinely partial genes or the result of incomplete sequence data, we sought to analyse the transcription and the genomic arrangement of a representative subset (Figure 2). TVAG_275420 is an ORF that encodes a predicted 478 aa protein of the ABCA subfamily that includes a TMD, a Walker A motif and a Signature sequence shortly followed by an in-frame TGA stop codon (Table S2, Figure 2A). This predicted protein is 70% identical to TVAG_440500, also an ABCA subfamily member (Table S2) of 830 aa that includes a full NBD. Downstream of TVAG_275420 there is a linked ORF, TVAG_275410 (Figure 2A, top panel) which is 82% identical to the last 70 amino acids of TVAG_440500. A Blastx search of the 232 bp intergenic region between TVAG_275420 and TVAG_275410 revealed that this is 88% identical to a similar region in the predicted TVAG_440500 protein sequence. Collectively, these data suggest that TVAG_275420, the intergenic region and TVAG_275410 are all part of a single gene/pseudogene (encoding a half ABC transporter) that is highly related to TVAG_440500. Moreover, an EST was found that matched the region from the 3′ end of TVAG_275420, the intergenic region and the complete sequence of TVAG_275410 (Figure 2A, open arrow). This finding suggests that there is either an error in the genomic sequence or that bicistronic transcription occurs at this locus. To assess this locus, we used RT-PCR to amplify putative transcripts running from TVAG_275420 through to TVAG_275410 (Figure 2D). Primers were designed to amplify any transcript or gDNA fragment from position 862 on TVAG_275420 to position 186 on TVAG_275410 (Table S1). We successfully amplified a band of the expected size at 990 bp by RT-PCR of polyA+ RNA (Figure 2D, lane 3). This band migrated at a similar size to one amplified from gDNA using the same primer set (Figure 2D, lane 2) but no such species was amplified from the negative control (Figure 2D, lane 4), confirming that the band in lane 2 (Figure 2B) originates from mRNA and not from gDNA contamination of the poly A+ template.

As a positive control, we ran a parallel set of reactions on the locus of TVAG_470720 (Figure 2C), an *abc* gene which is known to be expressed, based upon both EST and protein detection (our unpublished data). This gene encodes a complete TMD-NBD and both its transcript and a corresponding gDNA fragment were amplified using primers listed in Table S1 to yield bands of 1 kb (Figure 2D, lanes 8–10). The sequences of both the transcript and the gDNA fragment were found to be identical to the TVAG_470720 sequence from TrichDB. However, upon sequencing of both the gDNA and the amplified cDNA from the TVAG_275420/410 locus, we noted that a cytosine (C) was absent from a predicted C triplet at position 1414 to 1416 on the TrichDB TVAG_275420 sequence. The absence of this C residue results in an ORF of 1878 bp that runs from TVAG_275420 through to the 3′ end of TVAG_275410. Thus, it appears that a sequencing error, and not bicistronic transcription, is responsible for the original arrangement of genes shown in Figure 2A, top panel, and raises the possibility that the same may apply to other loci containing split *abc* genes.

We therefore investigated an additional locus (Figure 2B) that consists of TVAG_072420, an 875 bp ORF encoding a single predicted TM helix and TVAG_072410, a 1703 bp encoding five further TM helices followed by a complete NBD (Table S2). The two ORFs are separated by only 5 bp and no ESTs have been matched to either sequence. We designed primers to run from position 658 on TVAG_072420 to position 391 on TVAG_072410 (Table S1), thus expecting an amplicon of 613 bp. We were unable to amplify a band of the correct size by RT-PCR of poly A+ RNA but the same primers yielded a 0.6 kb band from gDNA (Figure 2D, lanes 5–7) which exactly matched the TrichDB sequence, confirming that the two ORFs are indeed separated by a stop codon and a 5 bp intergenic region.

These data collectively show that not all split *abc* genes can be explained by sequencing discrepancies. Two further examples of this were analysed to reinforce the fact that ABC transporter homologous DNA sequences are located in the intergenic regions between closely separated partial ABC transporter reading frames. The two additional examples (Two TVAG_049010 & TVAG_049020, and TVAG_254080, TVAG_254070 & TVAG_254060) are shown in Figure 2E and F respectively. In the second locus, it appears that an *abc* gene has been split into four parts that each contain at least an ABC motif (Figure 2F). No ESTs were found in the current databases to match any of these genes or intergenic regions. TVAG_049010 is predicted to encode three TM helices and is followed by a 93 bp intergenic region and then a downstream ORF TVAG_049020 encodes two further TM helices and a complete NBD (Figure 2E). As observed above, the intergenic region has high sequence identity, as detected by BLASTx, to another putative ABCB gene TVAG_127410. Thus, in the absence of a stop codon at the end of TVAG_049010, this locus could potentially comprise a complete half-transporter. We successfully amplified a fragment of 1.3 kb from genomic DNA (data not shown) using primers from position 191 on TVAG_049010 to position 710 on TVAG_0490120 (Table S1), and found the sequence of the amplicon to exactly match that on TrichDB.

The TVAG_254080/70/60 locus (Figure 2F) contains ORF TVAG_254080 with two predicted TMS followed by an open intergenic region that could potentially encode 3 TMS that are 44% identical to those of ORF TVAG_299600, a member of the ABCA subfamily. Downstream, TVAG_254070 contains a Walker A motif, with TVAG_254060 containing the Signature sequence and Walker B motif, and these two ORFs are separated by a 27 bp intergenic region. We amplified and sequenced a 2 kb region of the genomic locus (data not shown) from position 125 on TVAG_254080 through to position 466 on TVAG_254060 and again found the sequence to be identical to the TrichDB sequence, confirming that the arrangement of the partial genes depicted in Figure 2F is correct.

### The absence of full length ABC transporters from *T. vaginalis*

We further noted another category of loci where ORFs are arranged tail to tail, separated by short intergenic regions as shown by two examples with three open reading frames each in Figure 3. In each case, the size of the assembled ORFs could constitute a full length ABC transporter. The locus in the top panel (Figure 3A) consists of two ORFs, TVAG_245200 and TVAG_245210, separated by only 27 bp and arranged head to tail. TVAG_245220 is arranged tail to tail with 245210 with an intergenic space of 258 bp. We set out to investigate whether the contigs at this locus had been correctly assembled and sequenced to ascertain that the *T. vaginalis* genome does not bear any genes for full-length ABC transporters as suggested by the data presented in Table S2. To do so, we used primers designed to give a positive result if in the first case, TVAG_245220 were reversed and in the second case, if both TVAG_245200 and TVAG_245210 were reversed (Figure 3A). It was found that a PCR product for the locus was generated with a single primer, 245220R2, which bound to the corresponding complementary strand on both TVAG_245220 and TVAG_245200 (data not shown). The sequence for this fragment was identical to that in the TrichDB database, demonstrating that the original ORF arrangement in Figure 3A, top panel, was correct. TVAG_245200 is transcribed as evidenced by a matching EST from the TrichDB database, indicating that the ORFs in this locus are unlikely to represent pseudogenes.

The locus comprising TVAG_415970, TVAG_415980 and TVAG_415990 represents a similar situation as the previously described locus except that there is no intergenic region between TVAG_415980 and TVAG_415990 that are just separated by a TGA stop codon (i.e. TVAG_415980 and TVAG_415990 could comprise an intact half-transporter, linked head-to-head with another half-transporter TVAG_415970; Figure 3B). Using a similar strategy as with the TVAG_245200-220 locus, we set out to verify the possibility that either TVAG_415970 or TVAG_415980 and TVAG_415990 may be reversed or whether the original arrangement is correct. A 2.8 kb fragment was generated by PCR with a single primer (data not shown), TVAG_415990R1, which similarly to the previous case bound to opposite strands on two tail to tail ORFs. Sequencing of this product revealed that the TrichDB arrangement was correct and that the sequence was identical to that in the database.

Given that ORFs TVAG_415980 and TVAG_415990 are separated just by one TGA stop codon, we pursued study of this locus to investigate the possibility of stop codon read-through. *T. vaginalis* was transfected with a plasmid construct, pTagVag2-415980/90 that contained a fragment comprising the TVAG_415980 ORF and the TVAG_415990 ORF, including the intervening TGA stop codon. This fragment had been cloned upstream of a double haemagluttinin (HA) tag, such that detection of a recombinant protein by an anti-HA antibody would only occur if read-through happened or if the TGA stop codon were processed post-transcriptionally. PolyA+-enriched RNA isolated from the transfectants enabled us to verify by RT-PCR that the gene cassette from pTagVag2-415980/90 was transcribed. We detected amongst other smaller bands, a 0.9 kb band of the expected size that was amplified from the reverse-transcribed polyA+ template (Figure 3C, *lane 5*). This band was of the same size as that obtained from pTagVag2-415980/90 plasmid DNA that was used as a positive control (Figure 3C, *lane 4*), but not from negative controls (Figure 3C, *lane 3*). To investigate translation, we analysed transfected cells by immunofluorescence microscopy with anti-HA antibody. A diffuse distribution of anti-HA signal was seen in fixed transfected cells (Figure 3E) as opposed to wild-type C1 cells (Figure 3D). These data provides tentative evidence for stop codon read-through, although further experimental work would be required to substantiate this.

### Absence of the ABCG and ABCC subfamilies in *T. vaginalis*

The ABCG sub-family sequences are distinguished from other ABC proteins by their “reversed topology” [28], i.e. the NBD is found at the N-terminus of the protein, whereas the C-terminus contains the TMD. The family also contains only half-transporters in organisms whose genomes have been sequenced to date (e.g. see [9]). Examination of the *T. vaginalis* genome's complement of half transporters reveals none with this altered topology. Furthermore, even though many of the 31 unclassified proteins contain either a single NBD or a single TMD, no 2 of these are genomically arranged in a manner compatible with them forming a complete ABCG transporter following stop codon read through or sequencing inconsistencies explored above. Similarly, we were unable to detect any members of the ABCC sub-family, which are commonly identified by the presence of a large additional N-terminal TMD containing (usually) 5 TM α-helices. This N-terminal extension was not found in any of the *Trichomonas* ABC transporter sequences, and none of these sequences more similar to the ABCC transporters than to the ABCB transporters of other parasite genomes (*E. histolytica, P. falciparum*, *G. lamblia*).

This absence of ABCC and ABCG transporters must reflect biological perspectives of the organism. The absence of ABCG proteins may correlate with the expansion of ABCA proteins. Although both families have members that are involved in the export of lipids and their derivatives, only the ABCA family has members that are believed to be importers [8], [29], and the proposal is that in *T. vaginalis* a requirement for lipid import (see next section) has driven the ABCA expansion. For the ABCC family, the absence of members may be a correlate of the absence of a glutathione system in *T. vaginalis* [30] as many characterised eukaryotic ABCC members are either co-transporters of glutathione, or even transport directly GSH-conjugated substrates. The other members of the ABCC family function as ion channels or ion channel regulators (CFTR/ABCC6 and SUR/ABCC8,C9 respectively) which are absence from other early diverging eukaryotes [31].

### Expansion of the ABCA subfamily in *T. vaginalis*

The ABCA subfamily was the largest identified in *T. vaginalis*, with 34 putative transporters, several of which are transcribed as evidenced by expressed sequence tags. With the exception of two partial sequences, all have a (TMD-NBD) topology, range in length from 478–919aa and all bar 7 members of this subfamily have the characteristic extracellular loop (ECL) of the ABCA subfamily between their first and second predicted TM helices (Table S2). *Trichomonas vaginalis* has a severely compromised ability to synthesis lipids [32], and is therefore reliant on their import - a trait shared by *G. lamblia*, another species in which the ABCA proteins form a significant proportion (68%) of the ABC family (Table 2). Given that ABCA transporters in humans have been implicated in the export *and import* of a variety of lipids and lipid conjugates [29], [33] it is plausible that some ABCA transporters have evolved in *T. vaginalis* as *importers* of lipids rather than *exporters*.

**Table 2:** The classified ABC proteins of *T. vaginalis* compared with other eukaryotes.

Species	Number of proteins in each ABC sub-family								Total number of ABC proteins
	A	B	C	D	E	F	G	H/I	
*T. vaginalis*	34	27	0	2	1	3	0	31	98
*H. sapiens*	12	11	12	4	1	3	5	0	48
*D. discoideum*	11	9	14	3	1	4	21	5	68
*S. cerevisiae*	0	4	7	2	1	5	10	2	31
*G. lamblia*	15	0	4	0	1	1	0	1	22
*P. falciparum*	1	7	2	0	1	2	1	2	16
*E. histolytica*	2	7	7	0	1	2	2	3	26

**Figure 4:**
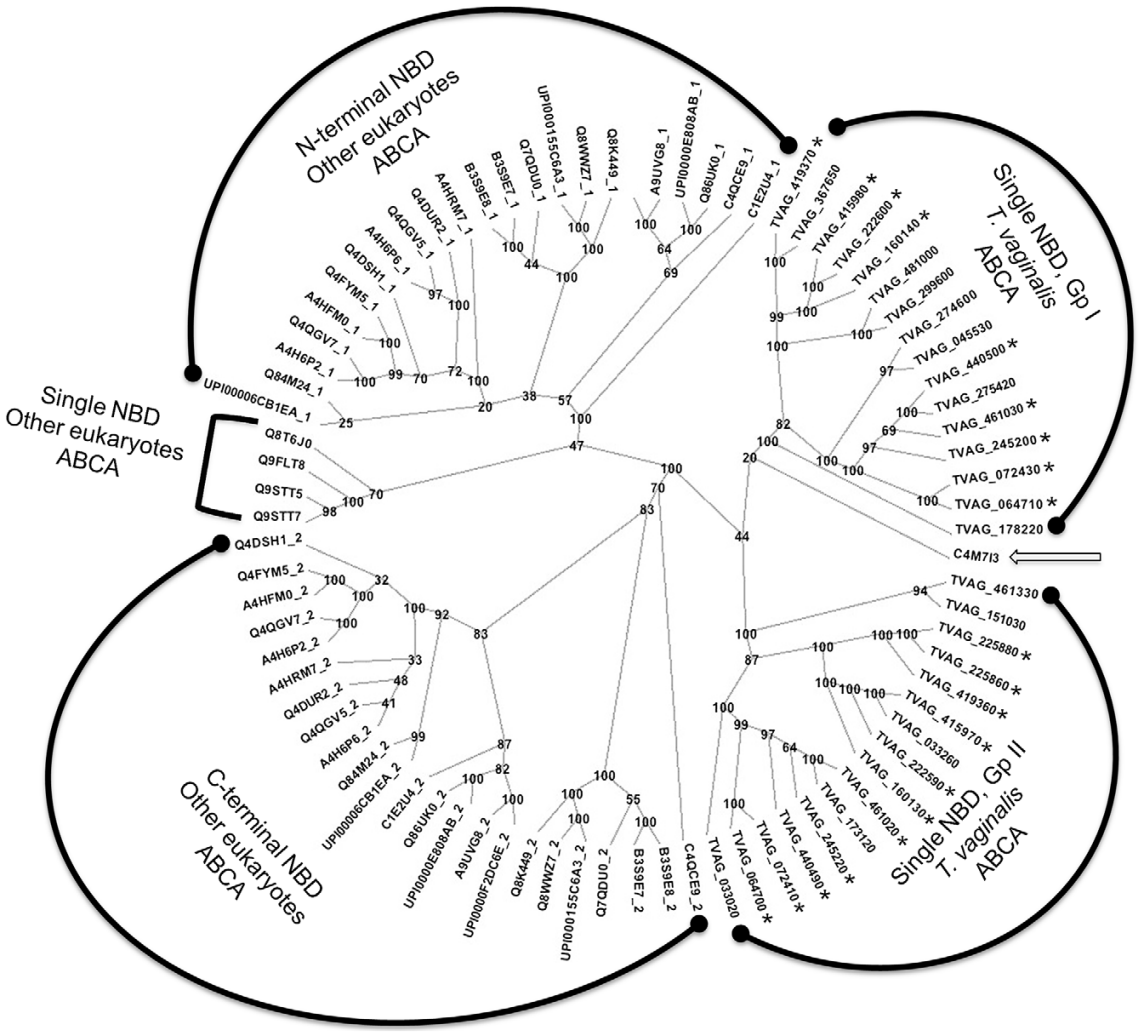
ABCA proteins in *T. vaginalis* and other eukaryotes show conservation of paired consensus and degenerate NBDs. Protein sequences for ABCA transporters from *T. vaginalis* and other eukaryotes had their TMD sequences removed, and in the case of full-length proteins, the sequence was bisected into N- and C-terminal halves. The alignment and boot-strapping were as described in Figure 1. The N-terminal NBD cluster contains almost entirely NBDs with consensus Walker and signature motifs, whereas the C-terminal cluster is almost always degenerate for at least one of the 3 motifs. For *T. vaginalis*, there are ten pairs of ABCA genes, and in all bar one case, the two members of each pair split on sequence into Group I (consensus) Walker/Signature) or Group 2 (degenerate Walker/Signature). *T. vaginalis* sequences marked with an asterisk also have an extracellular loop of 200–500 amino acids between the 1^st^ and 2^nd^ predicted TM helix as observed in many other eukaryotic ABCA proteins.

To date, ABCA transporters that have been characterised in other eukaryotes are full-length (i.e. 2 NBDs and 2 TMDs in the same polypeptide), unlike the transporters described here. In order to reconstruct the phylogenetic history for the *T. vaginalis* ABCA subfamily, we used sequences for TVAG_064700 and TVAG 064710 respectively as queries to search for homologues in UniProt. These two sequences were chosen as the genes are linked as inverted tandem repeats, and they both contain an ECL. Moreover, TVAG_064700 has a degenerate signature motif (LSDGD) whereas TVAG_064710 has a canonical one (LSGGQ). To be able to properly align the half-transporters from *T. vaginalis* to other eukaryotic full-length transporters, we selectively extracted NBD sequences from the latter to comprise an N-terminal and a C-terminal NBD region each. As shown in Figure 4, these ABCA NBD sequences from other eukaryotes form two distinct clusters, suggesting an early duplication event followed by divergence. It is of note that the C-terminal NBD almost always contains a degenerate signature motif in other eukaryotes whereas the N-terminal NBD invariably contains the canonical LSGGQ. The *T. vaginalis* ABCA single NBD sequences mimic the clustering of NBD sequences from other eukaryotes in that they too fall into two distinct groups termed Group I and Group II (Figure 4), with Group I sequences invariantly bearing a canonical signature motif and Group II sequences invariantly bearing a degenerate signature motif. Twenty of the *abca* genes are linked as pairs that appear to be inverted repeats (TrichDB and Table S2). Any one member of each pair (except for TVAG_225860 and TVAG_225880) has higher sequence similarity to other ORFs in the same group than to its linked repeat. For instance, TVAG_072430 and TVAG_064710 cluster together in a group that is distinct from another that includes their respective linked partners TVAG_072410 and TVAG_064700 (Figure 4). A possible scenario consistent with this data that accounts for the history of *abca* genes in eukaryotes is that ancestral *abca* genes existed both as half-transporters and full-transporters, prior to the evolution of the progenitor of *T. vaginalis* [34]. This lineage lost the full-length transporter gene, but the evolutionary forces that maintain the paired canonical and degenerate NBDs in full-length eukaryotic ABCA transporters are clearly still acting on the *Trichomonas abca* half-transporter genes. In the case of the 2258 locus, the duplicate locus has apparently duplicated again to give rise to two sets of inverted genes. This appears to be a recent duplication as the sequences of corresponding gene fragments are almost identical to each other.

### *T. vaginalis* ABCD-type proteins are localized to the ER

*T. vaginalis* is the only parasite in Table 2 encoding ABCD type transporters, with 2 half transporters, TVAG_470720 (556aa) and TVAG_605460 (546aa), similar to the total number found in other eukaryotic species [35], indicative of a lack of duplication (conversely to the above). The hypothetical *T. vaginalis* ABCD transporters are half transporters with the topology TMD-NBD, in common with all other non-plant eukaryotic ABCD proteins [9]. The majority of ABCD transporters have been localised to peroxisomes, where they are implicated in the transport of VLCFAs and other co-enzyme A conjugates into the peroxisome for β-oxidation.

**Figure 5:**
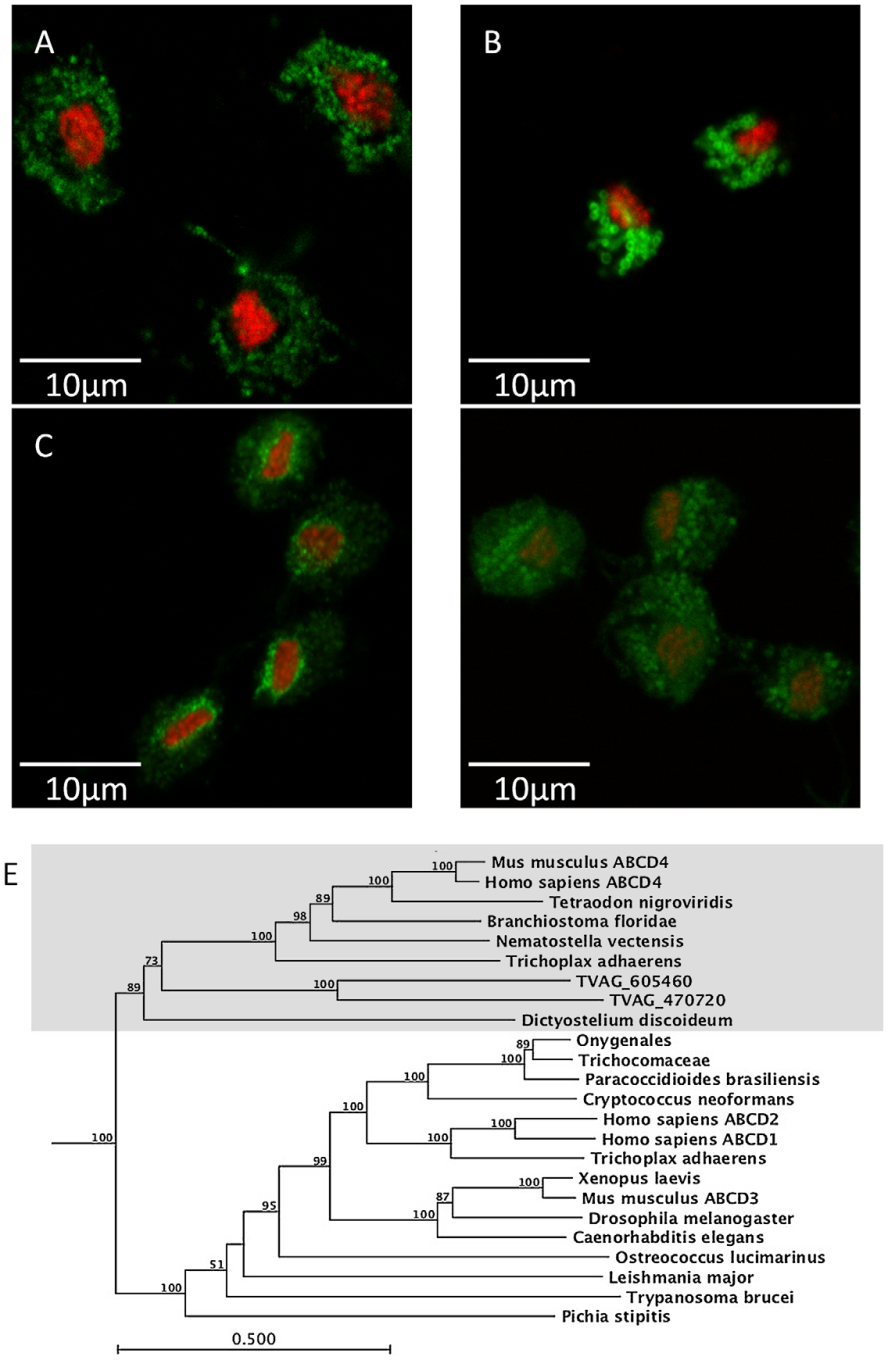
Localization of ABCD transporters in *T. vaginalis*. Parasites were fixed, labelled and imaged as described in the legend to Figure 3. The endoplasmic reticulum protein BiP in C1 (**A**) was detected with primary anti-BiP antibody, whilst the hydrogenosomal protein TOM40-3 (**B**) and two ABCD transformants (TVAG_470720; **C**; and TVAG_605460; **D**) were detected by reactivity to anti-HA antibodies. The distributions of BIP, TVAG_605460, and TVAG_470720 are all similar with a dispersed pattern of staining. In contrast, the hydrogenosome membrane protein TOM 40-3 (**B**) was detected in discrete spherical organelles, consistent with the size and shape of the hydrogenosome. **E** the ABCD transporters of eukaryotes for two distinct sub-families, one of which (grey boxed) has independent evidence to support endoplasmic reticulum localization, whereas the other sub-family are peroxisomal localized.

To investigate their sub-cellular localization, ABCD transporters TVAG_470720 and TVAG_605460 were expressed in *T. vaginalis* C1 cells with a C-terminal double haemagluttinin epitope, and were visualised using immunofluorescence microscopy (Figure 5). Control C1 cells were incubated with PI to detect the nucleus, and with anti-BiP antibody to detect the diffuse ER (Figure 5A). This contrasts directly with an alternative organelle membrane marker - a hydrogenosomal TOM40 homologue [36], which showed dozens of internal, discrete, vesicular structures with dimensions consistent with those of the hydrogenosome (Figure 5B; [2]). Both the ABCD transformants showed a highly diffuse distribution similar to that observed with BiP (Figure 5C, D) with TVAG_470720 also showing some additional perinuclear signal. Attempts to demonstrate co-localization with anti-BiP were confounded by this extreme diffuseness. Our argument for the ABCD transporters being localized to the ER is further supported by both the absence of peroxisomes from *T. vaginalis*, and by recent localization of a subset of ABCD proteins to the ER rather than to the peroxisome in humans and mice [37]. Notably, both TVAG_470720 and TVAG_605460 belong to this latter subset, rather than to the “classical” peroxisomal ABCD proteins (Figure 5E).

### Expansion of the ABCB subfamily in *T. vaginalis*

Twenty-seven proteins constitute the hypothetical ABCB subfamily in *T. vaginalis*, the second largest subfamily, comprising a significant proportion (28%) of all ABC proteins - a characteristic shared by *P. falciparum* (44%) and *E. histolytica* (27%). Such findings reflect the importance of ABCB transporters in these parasites and raise the possibility of ABCB-specific gene amplification having occurred. All hypothetical ABCB proteins have the same TMD-NBD topology as ABCA transporters, but lack the defining extracellular loop of the latter family, and range in length from 477–733aa. Additionally, and distinct from ABCA transporters, the ABCB members all contain consensus signature sequences, with a single exception.

In humans, ABCB proteins, both full transporters at the plasma membrane and half transporters dimerising intracellularly, have been implicated in various roles ranging from drug resistance (ABCB1 or MDR1) to peptide transport into the ER (ABCB2 and B3) and iron homeostasis in mitochondria (ABCB6) [7]. In *T. vaginalis*, the closest sequence to mammalian ABCB1/P-glycoprotein is TVAG_542450 (*Tvpgp1*; [27]), however research has not supported an involvement of *Tvpgp1* in resistance to metronidazole [27]. For other eukaryotic ABCB transporters, including Atm1, convincing homologues in *T. vaginalis* could not be identified by sequence analysis alone and further localization and functional studies will be required.

The evolution of the ABCB family in *T. vaginalis* was examined by constructing phylogenetic trees employing the same criteria as applied to the ABCA sequences above, i.e. eukaryotic full length transporters had their NBD sequence extracted and these were then aligned and neighbour-joining trees generated using bootstrap analysis (Figure S1). A similar conclusion to that regarding the *T. vaginalis* ABCA proteins is reached, i.e. that despite the absence of full length ABCB transporters, the proteins form two distinct clusters which mimics the N- and C-terminal NBDs of full length eukaryotic ABCB proteins, suggesting that evolutionary pressure has acted on the *T. vaginalis* half transporters as it has on the full transporters.

### Non-transporting ABCE and ABCF proteins

**Figure 6:**
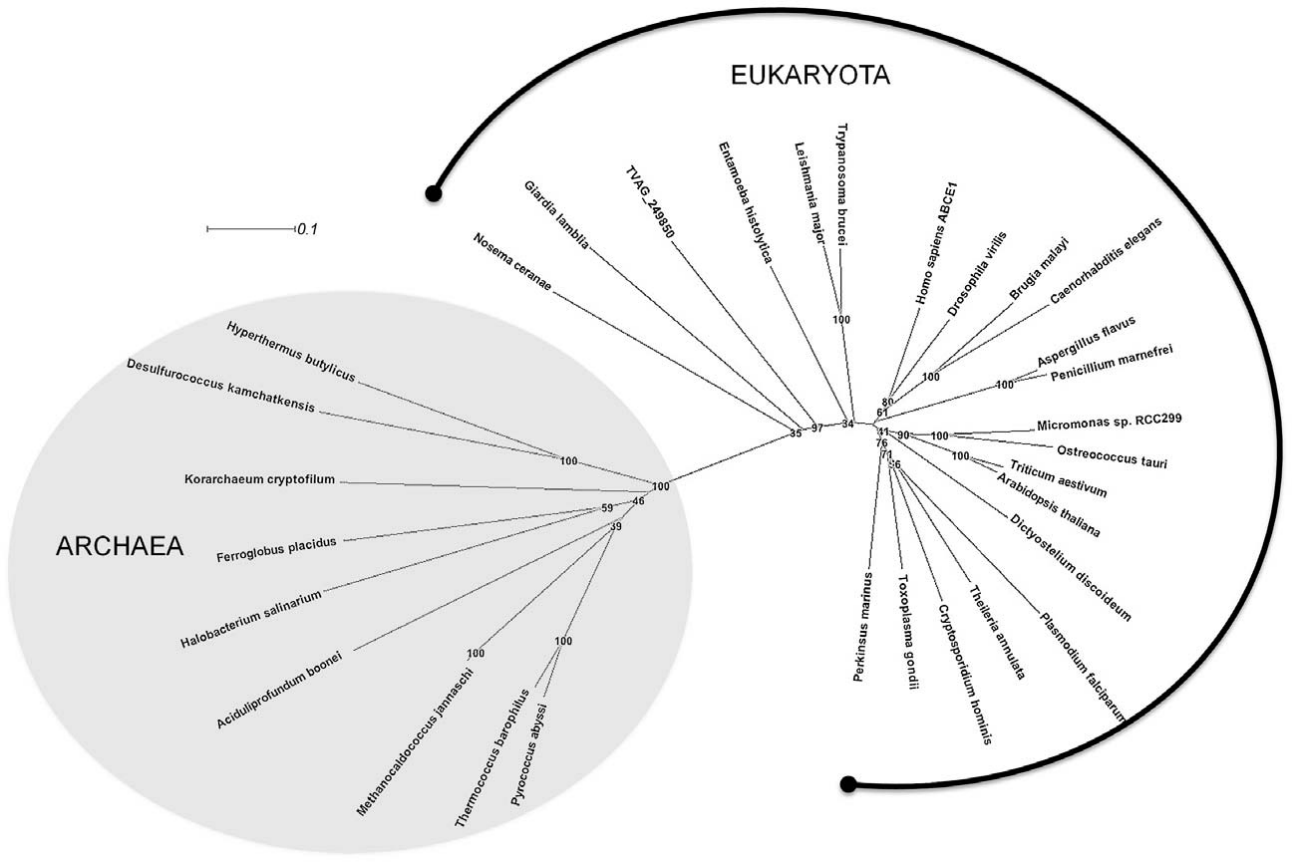
The ABCE proteins of archaea and eukaroytes. Multiple sequence alignment and boot-strapping was performed as described in the material and methods. The boot-strap values (percentages) are displayed on all branches.

ABCE sequences are absent from the eubacteria but present in all Archaea and eukaryotes for which genomic sequencing information is relatively complete. As expected, *T. vaginalis* contains a single homologue of human ABCE, and of all the ABC proteins the certainty that can be ascribed to TVAG_249850 as being ABCE is highest. *T. vaginalis* ABCE is 54–57% identical at the amino acid sequence level to other eukaryotic ABCEs, and 39–46% identical to those from Archaea (Figure 6). This degree of conservation is remarkable, to date only Hsp70 has been shown to have a similar level of conservation to homologues in both eukaryotes and Archaea [26], [38]. In spite of a structural description of ABCE [39] a complete understanding of the function of ABCE remains unresolved, although roles in translational control, ribosome assembly, and ribosome recycling [40], have been proposed. Clearly, its sequence conservation across the eukarya and Archaea argues for a function critical to the evolution of cell biology in these kingdoms [26].

*T. vaginalis* contains a larger number of predicted ABCF proteins than other parasites listed in Table 2. Consistent with other species' ABCF proteins, the hypothetical *T. vaginalis* ABCF subfamily is another group of non-transporters composed of two fused NBD domains (NBD_2_) and lacking membrane-spanning regions. Taxonomic BLAST searches with TVAG_427530 highlight the high level of conservation shown by the predicted *T. vaginalis* ABCF proteins, with E values as low as 9e-112 and identity as high as 42% with sequences from other species. Similar analysis with TVAG_385840 indicate that this is the *Trichomonas* homologue of yeast GCN20, an activator of eukaryotic translational initiation factor 2α-kinase (eIF2α-kinase) [41], showing 35% sequence identity (p-value of 3.9e-88). Confirmation of the function of *Trichomonas* ABCF proteins in translational control awaits further experimentation.

### Summary

Our analysis of the ABC transporters in *T. vaginalis* demonstrates three key findings with broader impact for our understanding of the parasite's biology. The first is the absence of full length ABC transporters. This is unique in eukaryotes for which we have sufficient sequence data. All other species described as early branching (e.g. mosses), and others classified as Excavata contain full-length ABC transporter genes (see footnote to Table 1). The absence of these from *T. vaginalis*, taken together with our data here on the maintained sequence separation of the half-transporters in the ABCA and ABCB sub-families suggests that either the full-transporter gene was an early loss in the evolution of *T. vaginalis* from a common ancestor with other eukaryotes, or that gene fusion events that produced full length ABC transporters in other extant eukaryotes have not been selected for in *T. vaginalis*. Another finding is the putative suppression of stop codons by *Trichomonas*. The expression of the ABCA half-transporter TVAG_415980 and TVAG_415990 as a single protein warrants further investigation of the mechanism for this suppression and its frequency. Finally, our confocal microscopy work shows that sub-cellular localization studies in *T. vaginalis* are accessible enabling further proteomic classification of this organism.

## Supporting Information

Figure S1 **ABCB proteins in** ***T. vaginalis*** **and other eukaryotes show conservation of NBDs.** Protein sequences for ABCB transporters from *T. vaginalis* and other eukaryotes had their TMD sequences removed, and in the case of full-length proteins, the sequence was bisected into N- and C-terminal halves. The alignment and boot-strapping were as described in Figure 1. The N-terminal NBDs of eukaryotic full-length ABCB proteins cluster as sequentially distinct from the C-terminal NBDs. Despite their being no full-length ABCB proteins in *T. vaginalis* the ABCB sequences also cluster into two sub-groups.
(TIF)

Table S1 **List of primers used in this study.** All primers are written 5′ to 3′, with restriction sites encoded underlined.
(DOC)

Table S2 **Predicted** ***T. vaginalis*** **ABC proteins.** Gene identifications are from TrichDB. The predicted length of each primary sequence is given, in addition to predictions regarding the number of transmembrane (TM) segments, predicted topology, and the identification of classical ABC transporter sequence motifs. Abbreviation used: EST – expressed sequence tag.
(DOC)
