# Supplementary figures and images for: The ATP-Binding Cassette Proteins of the Deep-Branching Protozoan Parasite Trichomonas vaginalis
Source: PLoS Negl Trop Dis. 2012 Jun 19;6(6):e1693. doi: 10.1371/journal.pntd.0001693 (PMC3378599; doi:10.1371/journal.pntd.0001693)

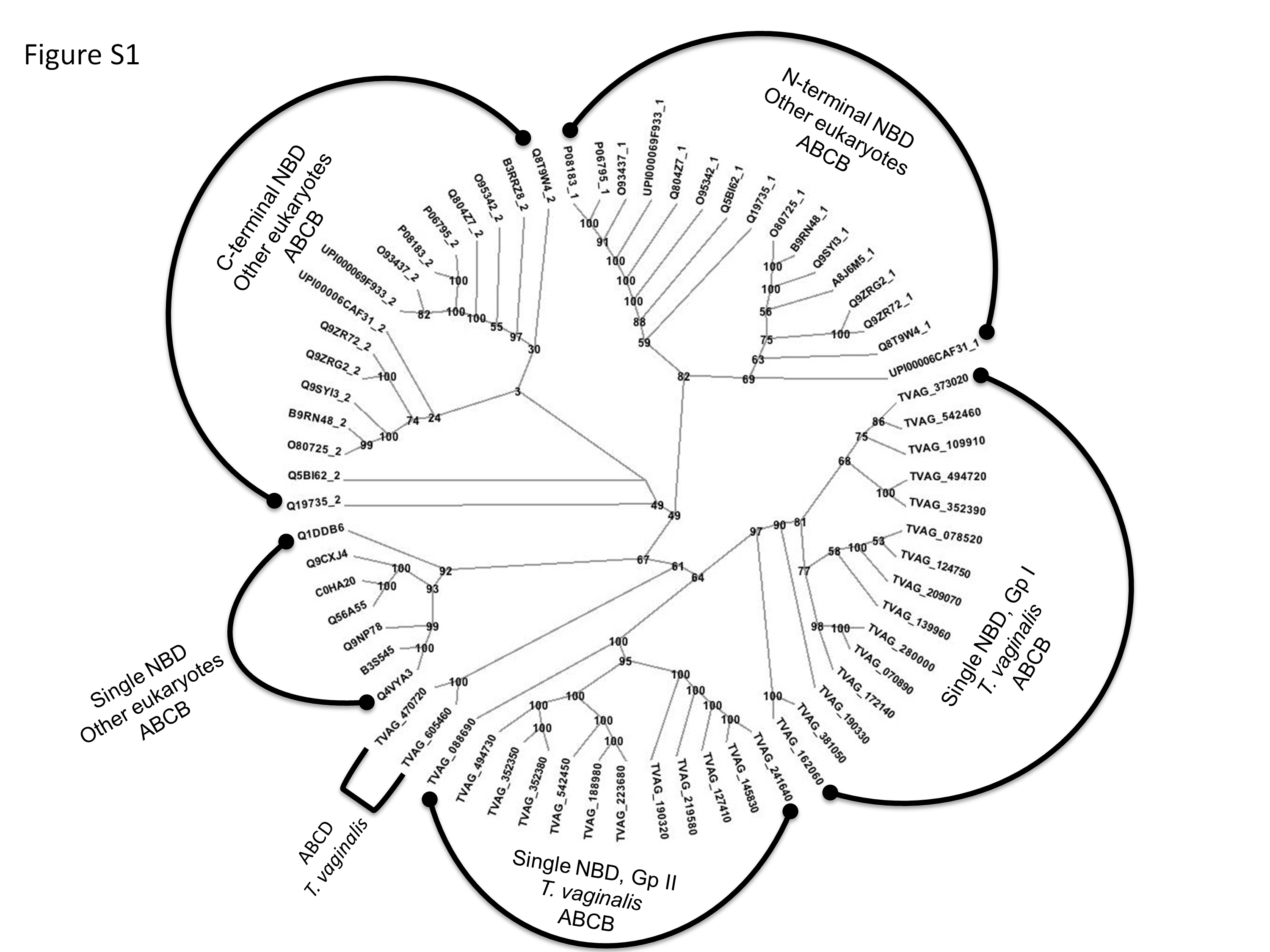

Supplement: Figure S1 — ABCB proteins in T. vaginalis and other eukaryotes show conservation of NBDs. Protein sequences for ABCB transporters from T. vaginalis and other eukaryotes had their TMD sequences removed, and in the case of full-length proteins, the sequence was bisected into N- and C-terminal halves. The alignment and boot-strapping were as described in Figure 1. The N-terminal NBDs of eukaryotic full-length ABCB proteins cluster as sequentially distinct from the C-terminal NBDs. Despite their being no full-length ABCB proteins in T. vaginalis the ABCB sequences also cluster into two sub-groups. (TIF) [file pntd.0001693.s001.tif]
