# Supplementary material for: The ATP-Binding Cassette Proteins of the Deep-Branching Protozoan Parasite Trichomonas vaginalis
Source: PLoS Negl Trop Dis. 2012 Jun 19;6(6):e1693. doi: 10.1371/journal.pntd.0001693 (PMC3378599; doi:10.1371/journal.pntd.0001693)
Supplement: Table S1 — List of primers used in this study. All primers are written 5′ to 3′, with restriction sites encoded underlined. (DOC) [file pntd.0001693.s002.doc]

Table S1: List of primers used in this study

| **Primer** | **Sequence** | **Restriction site**  **(* denotes naturally occurring)** |
| --- | --- | --- |
| 275420F1 | - GCTCTCGAGATCGTGGATTTGGTAATCAAC | - XhoI |
| 275410R1 | - GCTGGATCCTGTATCTTCTAATTGTTCCACAG | - BamHI |
| 072420F1 | - GCTGGATCCGGTTACTGCATCATGTACAGAG | - BamHI |
| 072410R1 | - GCTCTGCAGGCTTAGTTGAGTCACCATCAC | - PstI |
| 470720RTF | GCTGGATCCACATGCTGATGCATCGTTTAG | BamHI |
| 470720REV | GATCCTCGAGGGATCCATTGAGGGTATATCCACCCGC |  |
| 049010FOR | CGTGGTACCGTGACTTAACCTCCTCATTCG | KpnI |
| 049020REV | CCAACTTCTGCAGCCTTAGC | PstI* |
| 254080FOR | CGTCTGCAGGGTTTTCATTCTGTTCACTCGC | PstI |
| 254060REV | GCTGGTACCGAGGAATTTGGTCGCAAACAGGC | KpnI |
| 245200F2 | - GCTGGTACCCACCAGTCAAATGGGGAGATG | - KpnI |
| 245200R1 | - GCTGGTACCCATCTCCCCATTTGACTGGTG | - KpnI |
| 245220F1 | - GCATCAGCTGCTCAACCATATTC | - PvuII* |
| 245220R2 | - GTTGAGCAGCTGATGCATTCTTG | - PvuII* |
| 415970F1 | - GCTGGTACCTTACATGATATCATGTGGACTCTC | - KpnI |
| 415970R2 | - GCTCAGCTGGAGAGTCCACATGATATCATGTAA | - PvuII |
| 415980F2 | - GCTGGTACCTGGATTCGGCCAAAGGATC | - KpnI |
| 415990R1 | - GCTCAGCTGGATTTCATCCAATCAAGCAACTC | - PvuII |
| 415980F1 | - GCTGGATCCTCTACCCCTGTTCATGCTC | - BamHI |
| HAHA_RTR | - CTGGAACATCGTATGGGTAGGATCC | - BamHI |
| 415980NdeI | - GCTCATATGTCTATCAAAGTAGACAAGAAAGAG | - NdeI |
| 415990BamHI | - GCTGGATCCCACGTTTTCATCATCCACATAC | - BamHI |
