# Supplementary material for: The ATP-Binding Cassette Proteins of the Deep-Branching Protozoan Parasite Trichomonas vaginalis
Source: PLoS Negl Trop Dis. 2012 Jun 19;6(6):e1693. doi: 10.1371/journal.pntd.0001693 (PMC3378599; doi:10.1371/journal.pntd.0001693)
Supplement: Table S2 — Predicted T. vaginalis ABC proteins. Gene identifications are from TrichDB. The predicted length of each primary sequence is given, in addition to predictions regarding the number of transmembrane (TM) segments, predicted topology, and the identification of classical ABC transporter sequence motifs. Abbreviation used: EST – expressed sequence tag. (DOC) [file pntd.0001693.s003.doc]

Table S2: Predicted *T. vaginalis* ABC proteins

| Subfamily | Gene ID | Size/aa | EST/  Protein | No. of TM | Predicted topology | NBD Motifs | | |
| --- | --- | --- | --- | --- | --- | --- | --- | --- |
|  |  |  |  |  |  | Walker A | Walker B | Signature Sequence |
| ABCA | TVAG_033020 | 839 |  | 7-9 | TMD-NBD | GANGAGK | IILLDE | LSAGD |
|  | TVAG_033260 | 526 |  | 4-5 | TMD-NBD | ESNGAGK | TILLDE | MSGGN |
|  | TVAG_045530 | 808 |  | 7-9 | TMD-NBD | GPNGAGK | LVIMDE | LSGGQ |
|  | TVAG_064700 | 861 |  | 6-8 | TMD-NBD | GANGAGK | IILLDE | LSDGD |
|  | TVAG_064710 | 833 |  | 5-7 | TMD-NBD | GPNGAGK | IIMMDE | LSGGQ |
|  | TVAG_072410 | 568 |  | 5-6 | TMD-NBD | GANGAGK | IILLDE | LSDGD |
|  | TVAG_072430 | 775 |  | 5-7 | TMD-NBD | GPNGAGK | IIMMDE | LSGGQ |
|  | TVAG_117420 | 183 | EST | 0 | NBD | - | IIVLDE | - |
|  | TVAG_151030 | 757 |  | 7-8 | TMD-NBD | GANGAGK | FLILDE | LPPPL |
|  | TVAG_160130 | 640 |  | 6-7 | TMD-NBD | GSNGAGK | TILLDE | MSGGN |
|  | TVAG_160140 | 768 |  | 6-8 | TMD-NBD | GPNGSGK | LVILDE | LSGGQ |
|  | TVAG_173120 | 919 |  | 7-9 | TMD-NBD | GANGAGK | IILLDE | LSEGD |
|  | TVAG_178220 | 785 |  | 7 | TMD-NBD | GPNGSGK | IIVLDE | LSGGQ |
|  | TVAG_222590 | 817 |  | 7-8 | TMD-NBD | GSNGAGK | TILLDE | MSGGN |
|  | TVAG_222600 | 716 |  | 6-7 | TMD-NBD | GPNGSGK | LVILDE | LSGGQ |
|  | TVAG_225860 | 805 |  | 6-8 | TMD-NBD | GSNGAGK | IILLDE | MSGGN |
|  | TVAG_225880 | 799 |  | 7-9 | TMD-NBD | GSNGAGK | IILLDE | MSGGN |
|  | TVAG_245200 | 648 | EST | 7-9 | TMD-NBD | GPNGAGK | IIMMDE | LSGGQ |
|  | TVAG_245220 | 762 |  | 6-8 | TMD-NBD | GANGAGK | IILLDE | LSEGD |
|  | TVAG_274600 | 782 |  | 6-8 | TMD-NBD | GPNGAGK | IVVMDE | LSGGQ |
|  | TVAG_275420 | 478 | EST | 5-6 | TMD-NBD | GPNRAGK | IIMMDE | LSGGQ |
|  | TVAG_299600 | 531 |  | 3-4 | TMD-NBD | GPNGSGK | FVILDE | LSAGQ |
|  | TVAG_367650 | 777 | EST | 6-8 | TMD-NBD | GPNGSGK | FVILDE | LSGGQ |
|  | TVAG_415970 | 697 |  | 7-8 | TMD-NBD | GSNGAGK | TILLDE | MSGGN |
|  | TVAG_415980 | 478 |  | 6-7 | TMD-NBD | GPNGSGK | LVILDE | LSGGQ |
|  | TVAG_419360 | 827 |  | 6 | TMD-NBD | GCNGAGK | IILVDE | MSGGN |
|  | TVAG_419370 | 729 | EST | 7-9 | TMD-NBD | GPNGCGK | FVILDE | LSGGQ |
|  | TVAG_432310 | 297 |  | 0 | NBD | - | TILLDE | MSGGN |
|  | TVAG_440490 | 846 | EST | 6-8 | TMD-NBD | GANGAGK | IILLDE | LSEGD |
|  | TVAG_440500 | 830 |  | 7-8 | TMD-NBD | GPNGAGK | IIMMDE | LSGGQ |
|  | TVAG_461020 | 877 | EST | 6-8 | TMD-NBD | GANGAGK | IILLDE | LSEGD |
|  | TVAG_461030 | 832 | EST | 7-8 | TMD-NBD | GPNGAGK | IIMMDE | LSGGQ |
|  | TVAG_461330 | 761 | EST | 6-8 | TMD-NBD | GSNGAGK | IIVLDE | LSAGL |
|  | TVAG_481000 | 537 | EST | 4-5 | TMD-NBD | GPNGSGK | LVILDE | LSGGQ |
|  |  |  |  |  |  |  |  |  |
| ABCB | TVAG_070890 | 733 |  | 5-6 | TMD-NBD | GHSGSGK | ILLCDE | LSGGQ |
|  | TVAG_078520 | 710 |  | 3-5 | TMD-NBD | GHYGSGK | ILICDE | LSGGQ |
|  | TVAG_088690 | 597 |  | 6 | TMD-NBD | GESGCGK | ILLLDE | LSGGQ |
|  | TVAG_109910 | 618 |  | 4-5 | TMD-NBD | GHSGSGK | ILICDE | LSGGQ |
|  | TVAG_124750 | 713 | EST | 6 | TMD-NBD | GHSGSGK | ILICDE | LSGGQ |
|  | TVAG_127410 | 597 | EST | 5-6 | TMD-NBD | GESGCGK | ILLLDE | LSGGQ |
|  | TVAG_139960 | 532 |  | 3-4 | TMD-NBD | GHSGSGK | ILICDE | LSGGQ |
|  | TVAG_145830 | 589 |  | 4-5 | TMD-NBD | GESGSGK | IIILDE | LSGGQ |
|  | TVAG_162060 | 609 |  | 3-5 | TMD-NBD | GHSGSGK | VLITDE | LSGGQ |
|  | TVAG_172140 | 729 |  | 5-6 | TMD-NBD | GHSGSGK | ILLCDE | LSGGQ |
|  | TVAG_188980 | 595 |  | 3-5 | TMD-NBD | GESGCGK | ILLLDE | LSGGQ |
|  | TVAG_190320 | 597 |  | 5-6 | TMD-NBD | GESGCGK | ILLLDE | LSGGQ |
|  | TVAG_190330 | 613 |  | 5-6 | TMD-NBD | GKSGSGK | VFIFDE | LSGGQ |
|  | TVAG_209070 | 701 |  | 5-6 | TMD-NBD | GHSGSGK | ILLCDE | LSGGQ |
|  | TVAG_219580 | 597 |  | 5 | TMD-NBD | GESGCGK | ILLLDE | LSGGQ |
|  | TVAG_223680 | 535 |  | 3-5 | TMD-NBD | GESGCGK | ILLLDE | LSGGQ |
|  | TVAG_241640 | 600 |  | 4 | TMD-NBD | GESGSGK | IIILDK | LSGGQ |
|  | TVAG_280000 | 658 |  | 5-6 | TMD-NBD | GHSGSGK | ILLCDE | LSGGQ |
|  | TVAG_352350 | 587 |  | 5-7 | TMD-NBD | GESGCGK | ILLLDE | LSGGQ |
|  | TVAG_352380 | 605 |  | 6-7 | TMD-NBD | GESGCGK | ILLLDE | LSGGP |
|  | TVAG_352390 | 617 |  | 5 | TMD-NBD | GHSGSGK | ILITDE | LSGGQ |
| @ | TVAG_373020 | 555 |  | 3-4 | TMD-NBD | GHSGSGK | ILVCDE | VSGGQ |
|  | TVAG_381050 | 608 |  | 4 | TMD-NBD | GHSGSGK | ILITDE | LSGGQ |
|  | TVAG_494720 | 691 | EST | 5-6 | TMD-NBD | GHSGSGK | ILITDE | LSGGQ |
|  | TVAG_494730 | 477 |  | 4-5 | TMD-NBD | GESGCGK | - | LSGGQ |
|  | TVAG_542450 | 571 | EST | 5 | TMD-NBD | GESGCGK | ILLLDE | LSGGQ |
|  | TVAG_542460 | 698 | EST | 5 | TMD-NBD | GHSGSGK | ILICDE | LSGGQ |
| ABCDq | TVAG_470720 | 556 | EST/PRT | 5-6 | TMD-NBD | GPSGAGK | FALLDE | LSPGE |
|  | TVAG_605460 | 546 | EST | 3-5 | TMD-NBD | GPSGAGK | FALLDE | LSPGE |
| ABCE | TVAG_249850 | 600 | EST | 0-3 | NBD_2_ | GQNGIGK/  GQNGVGK | LYLIDE  LGIL | LSGGE  LSGGE |
| ABCF | TVAG_326670 | 712 |  | 0 | NBD_2_ | GRNGLGK  GANGIGK | VLMLDE  VMLLDE | LSGGQ  - |
|  | TVAG_385840 | 705 |  | 0-1 | NBD_2_ | GRNGMGK  GANGSGK | VLMLDE  LIIMDE | LSGGQ  - |
|  | TVAG_427530 | 691 |  | 0 | NBD_2_ | GRNGMGK  GGNGAGK | VLLLDE  LLLLDE | LSGGQ  LSGGF |
| Others | TVAG_006580 | 116 |  | 0 | NBD | - | IILLDE | MSGGN |
|  | TVAG_006590 | 353 |  | 0 | NBD | GPNGSGK | FVILDE | LSGGN |
|  | TVAG_006600 | 376 |  | 0-1 | NBD | GANGAGK | IILLDE | MSGGN |
|  | TVAG_072420 | 292 |  | 1 | TMD | - | - | - |
|  | TVAG_024550 | 345 |  | 2-3 | NBD | GPNGSGK | LVILDE | LSGGQ |
|  | TVAG_049010 | 210 |  | 3 | TMD | - | - | - |
|  | TVAG_049020 | 360 |  | 2-3 | NBD | GESGCGK | ILLLDE | LSGGQ |
|  | TVAG_109920 | 279 |  | 4-6 | TMD | - | - | - |
|  | TVAG_109930 | 149 |  | 0 | NBD | - | ILLLDE | LSGGQ |
|  | TVAG_136030 | 283 |  | 0 | NBD | GPNGAGK | LVIMNE | VSGGQ |
|  | TVAG_147100 | 464 |  | 2 | NBD | GHSGSGK | ILICDE | VSGGQ |
|  | TVAG_147110 | 188 |  | 2 | TMD | - | - | - |
|  | TVAG_208810 | 288 |  | 0 | NBD | - | ILLLDE | LSGGD |
|  | TVAG_218950 | 348 |  | 0-2 | NBD | GPNGSGK | LVILDE | LSGGQ |
|  | TVAG_225870 | 358 | TAA | 0-2 | NBD | GPNGCGK | IVILDE | LSGGQ |
|  | TVAG_225890 | 424 | TAG | 1-2 | NBD | GPNGCGK | IVILDE | LSGGQ |
|  | TVAG_243600 | 165 |  | 0 | NBD | - | LLILDE | LSTGN |
|  | TVAG_248260 | 405 | EST | 1-2 | NBD | GPYSSGK | IVILDE | LSGGT |
|  | TVAG_254060 | 195 |  | 0-1 | NBD | - | LVILDE | LSAGQ |
|  | TVAG_254070 | 151 |  | 0-1 | NBD | GPNGSGK | - | - |
|  | TVAG_275410 | 71 | EST | 0 | partial NBD |  |  |  |
|  | TVAG_302430 | 126 |  | 0 | NBD | HSGSGKS | - | - |
|  | TVAG_352360 | 416 |  | 5 | TMD | GHSGSGK | - | - |
|  | TVAG_352370 | 169 |  | 0 | NBD | - | ILITDE | LSGGQ |
|  | TVAG_353200 | 475 |  | 1-3 | NBD | GHSGCGK | LLITDE | LSGGQ |
|  | TVAG_353220 | 438 |  | 2-3 | NBD | GPNGSGK | FVILDE | LSGGN |
|  | TVAG_364970 | 275 |  | 0-1 | NBD | - | LILLDE | MSGGN |
|  | TVAG_432320 | 432 |  | 2-4 | NBD | GPNGSGK | LVLLDE | LSGGQ |
|  | TVAG_461460 | 206 |  | 0-1 | NBD | - | IILLDE | MSGGN |
|  | TVAG_478620 | 424 |  | 1-2 | NBD | GANGAGK | IVLLDE | LSGGN |
|  | TVAG_487000 | 449 |  | 6 | TMD | GHSGSGK | - | - |
|  | TVAG_542470 | 251 |  | 0 | NBD | GESGCGK | ILLLDE | LSGGQ |
